# Etiologies of Acute Undifferentiated Febrile Illnesses in and near Iquitos from 1993 to 1999 in the Amazon River Basin of Peru

**DOI:** 10.4269/ajtmh.22-0259

**Published:** 2022-09-26

**Authors:** Douglas M. Watts, Kevin L. Russell, Mark T. Wooster, Trueman W. Sharp, Amy C. Morrison, Tad J. Kochel, Christian T. Bautista, Karla Block, Carolina Guevara, Patricia Aguilar, Pedro M. Palermo, Carlos Calampa, Kevin R. Porter, Curtis G. Hayes, Scott C. Weaver, Amelia Travassos de Rosa, Joseph M. Vinetz, Robert E. Shope, Eduardo Gotuzzo, Hilda Guzman, Robert B. Tesh

**Affiliations:** ^1^ U.S. Naval Medical Research Unit No. 6, Lima, Peru;; ^2^ University of California, Davis School of Veterinary Medicine Department of Pathology, Microbiology, and Immunology, Davis, California;; ^3^ Peruvian Ministry of Health, Loreto Health Subregion, Iquitos, Peru;; ^4^ U.S. Naval Medical Research Center, Silver Spring, Maryland;; ^5^ World Reference Center for Emerging Viruses and Arboviruses University of Texas Medical Branch, Galveston, Texas;; ^6^ Department of Medicine, Universidad Peruana Cayetano Heredia, Lima, Peru.

## Abstract

The objective of this study was to determine the etiology of febrile illnesses among patients from October 1, 1993 through September 30, 1999, in the urban community of Iquitos in the Amazon River Basin of Peru. Epidemiological and clinical data as well as blood samples were obtained from consenting patients at hospitals, health clinics and private residences. Samples were tested for arboviruses in cell cultures and for IgM and IgG antibodies by ELISA. Blood smears were examined for malaria, and sera were tested for antibodies to *Leptospira *spp. by ELISA and microscopic agglutination. Among 6,607 febrile patients studied, dengue viruses caused 14.6% of the cases, and Venezuelan equine encephalitis virus caused 2.5%, Oropouche virus 1.0%, Mayaro virus 0.4%, and other arboviruses caused 0.2% of the cases. Also, 22.9% of 4,844 patients tested were positive for malaria, and of 400 samples tested, 9% had evidence of acute leptospirosis. Although the study was not designed to assess the importance of these pathogens as a cause of human morbidity in the total population, these results indicate that arboviruses, leptospirosis, and malaria were the cause of approximately 50% of the febrile cases. Although the arboviruses that were diagnosed can produce asymptomatic infections, our findings increased the overall understanding of the relative health burden of these infections, as well as baseline knowledge needed for designing and implementing further studies to better assess the health impact and threat of these pathogens in the Amazon Basin of Peru.

## INTRODUCTION

Arboviruses are a significant cause of morbidity and mortality among human and domestic animal populations throughout the world.^1^ More than 500 viruses, including the arthropod-borne viruses, and some that have not been associated with an arthropod vector are classified into five major taxonomic families, including *Togaviridae*,* Flaviviridae*,* Bunyaviridae*,* Reoviridae*, and *Rhabdoviridae*.^1^^–^^3^ Approximately 100 are known to cause human disease, and approximately 40 infect domestic animals. The clinical manifestations of arboviral infections in humans are diverse but can be divided into three major syndromes: systemic febrile illnesses, encephalitis, and hemorrhagic fever.^1^^,^^2^ The most commonly syndrome is a mild-to-moderate, self-limited, undifferentiated acute febrile illness that may progress to a severe or life-threatening illness.

The impact of arboviral diseases on human health continues to grow, primarily because of increasing risk of infection.^1^ Some of the major contributing factors include growing urbanization related to the steadily increasing and expanding human population, increasing frequency and expansion of rapid air travel, human-associated uses and exploitation of the natural environment as well as the lack of or breakdown in vector-control programs. These factors have led to an increase in the abundance of the vector(s) by increasing larval habitats and by expanding the geographic range of both the viruses and the vectors.

The measures more commonly used to prevent arboviral diseases are those aimed at controlling the vectors.^1^ However, these approaches are difficult to establish and sustain because of the cost, human complacency, and/or ineffectiveness due to the resistance of arthropods to insecticides. Also, other methods, such as active surveillance of human and vector populations for infection as indicators for impending epidemics, and for emergency vector control measures are not usually affordable or feasible, especially in the underdeveloped regions of the world. Consequently, the expanding geographic pattern of arboviruses and associated arthropod vectors presents an increasing global health threat to human and animal populations.

Among the more important globally distributed human diseases that mimic syndromes caused by arboviruses are the parasitic disease, malaria and the bacterial disease, leptospirosis, both of which have specific treatments.^4^^,^^5^ The incidence of these diseases also continues to increase because of the extension of human activities into new areas, accompanied by novel exposures—for example, exploitation of Amazon rainforest for natural resources or jungle warfare training for military personnel. Approaches to prevent malaria include vector control and chemoprophylaxis. Leptospirosis can be prevented by weekly doxycycline therapy or by education to reduce exposure to infected rodent reservoirs, but there is no suitable vaccine for humans.^6^^,^^7^

Currently, more than 100 arboviruses are recognized in South America, and others remain to be identified and classified.^2^ Of these, the viruses associated more frequently with human disease, based mainly on periodic epidemics, are dengue (DEN), Venezuelan equine encephalitis (VEE), Oropouche (ORO), Mayaro (MAY), Rocio (ROC), and yellow fever (YF).^8^^–^^13^ Among these viruses, DEN, VEE, ROC, and YF have been associated with fatal disease. Except for focal areas of the Amazon rainforest, the epidemiology and geographic distribution of these viruses, as well as the likely possibility of many undescribed arboviruses, is largely unknown.^14^^,^^15^

The history of arboviral diseases in Peru began during the early half of the 19th century with the description of an outbreak of a disease resembling yellow fever during 1913 in the Amazon rainforest.^16^ Since then, annual outbreaks of yellow fever have continued to occur, with the largest sylvan outbreak ever recorded during 1995 in the Amazon rainforest along the Eastern foothills of the Andes Mountains.^17^ Epidemics and epizootics of VEE virus subtype IAB virus occurred among humans and equine at intermittent intervals from 1925 through 1973 along the Pacific coastal plains, extending southward from the most northern Department of Tumbes to Departments south of Lima.^9^^,^^18^^–^^20^ Other than for YF and VEE viruses, it was not until 1990–1991 that other arboviruses, including DEN-1 and ORO, were confirmed as the cause of human disease in Iquitos, Peru.^21^^,^^22^

Several other arboviruses, including St. Louis encephalitis (SLE), eastern equine encephalitis (EEE, recently reclassified as Madariaga [MAD] virus, group C), Marituba and Guama viruses, and VEE subtype viruses ID and IIIC were isolated during the early 1970s from either mosquitoes or sentinel hamsters in Quisto Cocha, a site about 5 km from Iquitos.^23^^–^^28^ Also, serological evidence of VEE infection among human and equine infection was demonstrated for some of these viruses during the 1960s and 1970s, but none were reported as the cause of illness.^29^^,^^30^

In 1993, the staff of the U.S. Naval Medical Research Institute Detachment (NAMRID), Lima, Peru, (renamed the U.S. Naval Medical Research Unit No. 6); the Peruvian Ministries of Health (MOH) and Defense, Iquitos, Peru; and the University of Texas Medical Branch (UTMB), Galveston, Texas, initiated a longitudinal active surveillance study in Iquitos to determine the arboviral etiology of febrile illnesses. This study was also conducted to describe the temporal distribution, frequency, clinical manifestations, and other epidemiological features of arboviral cases. Selected observations during this study, including descriptions of some of the MAY and VEE cases and studies that identified the subtypes of VEEV involved other collaborators from the U.S. Army Medical Research Institute of Infectious Diseases, Frederick, MD, and the Centers for Disease Control and Prevention were published previously.^31^^–^^33^ In addition, sera from a random sample of patients were tested for *Plasmodium* species and for leptospiral infections.

## MATERIALS AND METHODS

### Study site and study population.

**Figure 1.**
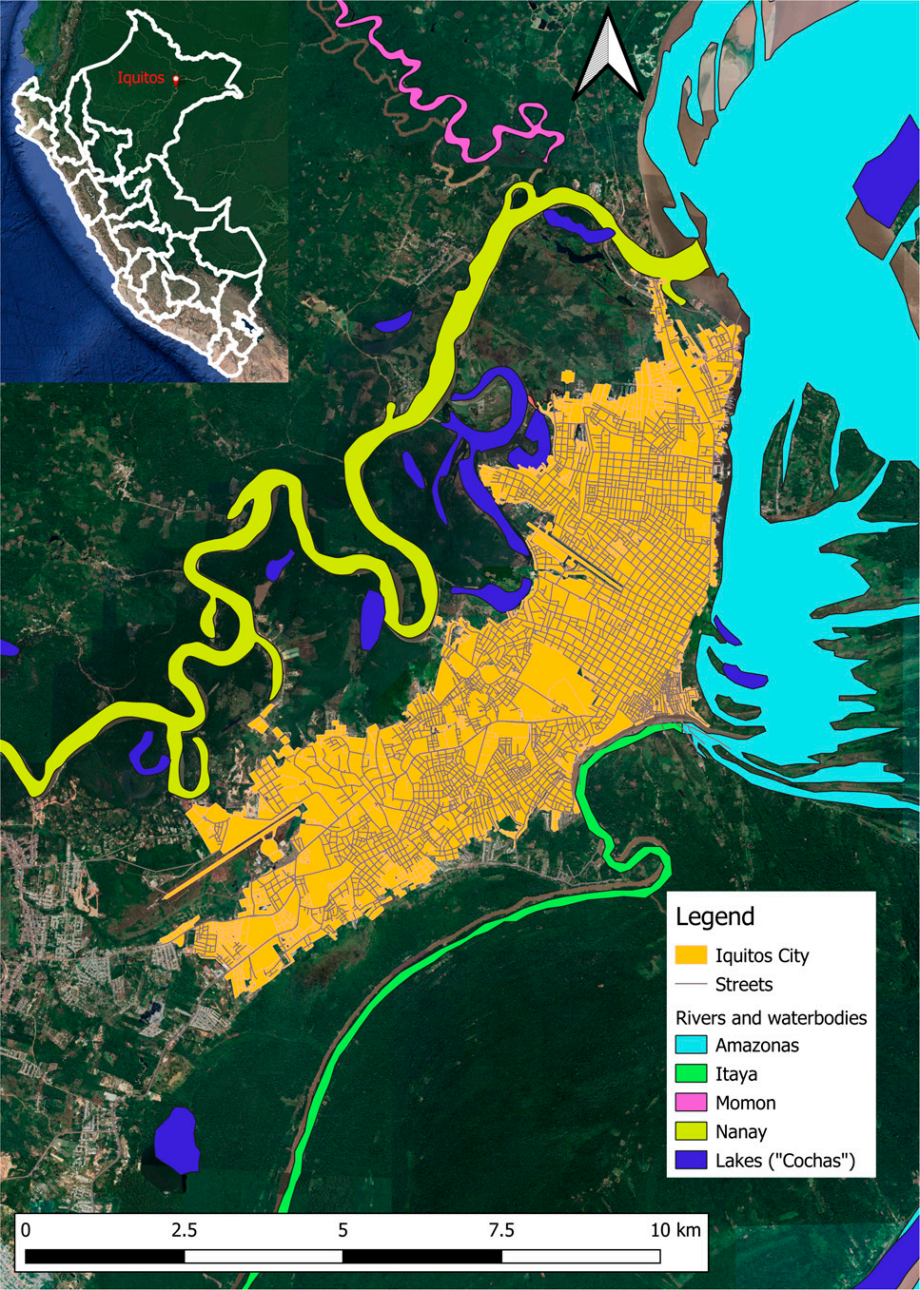
Locality of febrile disease study site in Iquitos, Peru.

The study was conducted in Iquitos, an urban community of 5,932 km^2^ located 120 m above sea level, 73.2 degrees west and 3.7 degrees south in the Amazon River Basin, Department of Loreto, Peru (Figure 1).^34^^,^^35^ Iquitos is a city with a population in 1993 of 274,000 surrounded by the Amazon River to the North, the Nanay River to the West and the Itaya River to the East. At the time of this study, the urban sector, a 4 to 5 km^2^ area, merges with periurban and rural populations and is surrounded by secondary rainforest. Except for the paved city hub area, most of the urban dwellings are located among a variety of fruit trees, shrubs, and grasses. The major occupations in Iquitos are housekeepers, military, agriculture, fishing, teaching, business merchants, and tourism.^34^^,^^35^ Overall, the climate in Iquitos is hot, humid, and rainy throughout the year, and even though there are no distinct seasons, rains are more frequent from October to May. The climate is tropical, with an average temperature of 25.5°C and an average mean annual precipitation of 2,840 mm (111.8 inches). The average temperature of the coldest month (July) is 26.3°C (79.3°F) and of the warmest month (November) is 27.6°C (81.8°F). The precipitation ranges from 155 mm (6.1 in) in the driest month (August) to 350 mm (13.8 in) in March, the wettest month. Most of the population is mixed Spanish and American Indian (Mestizo). Storage of water for household use in a variety of artificial containers is a common practice, thus providing larval habitats for *Aedes aegypti*, the mosquito vector of DENV. Several other genera of mosquitoes, including *Culex*,* Aedes*, and *Psorophora* inhabit the city.^36^ Local transportation includes bicycles, two- and three-wheel motorized vehicles, and automobiles. Transportation into and out of Iquitos is confined to boats and airplanes, including local and international planes. Locally produced vegetables, fruits, swine, and chickens, supplemented with fish from the Amazon, Nanay, and Itaya Rivers and wild plants, fruits, and animals, are the principal foods. The more affluent families supplement their diet with food shipped into Iquitos via plane from Lima, the capital city of Peru. Primary and secondary education is provided free, and there are two universities in Iquitos. Sources of water are a municipal piped system, the surrounding rivers, and rain. Infectious diseases are a major cause of health problems, the more common ones being parasitic diseases, gastroenteritis, malaria, tuberculosis, dengue, and other arboviral illnesses. Medical services are provided by two MOH hospitals (280 beds), one Army (180 beds) hospital, one Air Force (30 beds) hospital, one Navy (52 beds) hospital, one police (20 beds) hospital, and one missionary (40 beds) hospital and several MOH and private outpatient clinics. These medical care facilities are used by residents of Iquitos and surrounding village communities, as well as by military troops stationed in Iquitos and by troops deployed throughout the Amazon basin region. Most all patients who attended the outpatient clinics and/or were admitted to the hospitals are civilians.

### Study patients.

All patients who volunteered to participate in this study were enrolled under a human use protocol (DoD30516) titled the Ecology and Epidemiology of Dengue viruses in the Amazon Basin Region of Peru approved by the Peruvian Navy Hospital, Lima, Peru, the Peruvian MOH, Iquitos, Peru, the U.S. NMRC and the UTMB institutional review boards. All volunteer patients 18 years and older signed an informed consent form and patients under age 18 years either signed with parental approval or the parent or guardians signed on behalf of volunteer patients under 6 years. The initial approach, which extended from October 1993 through September 1994, was to enroll volunteer patients during the first 4 hours on Monday, Wednesday, and Friday of each week at four MOH outpatient clinics and at the two MOH and two Peruvian Military Hospitals. The surveillance sites for febrile cases were increased from October 1994 through September 1997 to include an additional eight outpatient clinics and visits to private residences by MOH workers. In October 1997, three more outpatient clinics were added for 19 permanent sites through September 1999. Enrollment at the additional outpatient clinics was conducted on Tuesday and Thursday. These increases in the number of surveillance sites were aimed at increasing the coverage of the population.

A standardized case definition was used by KB (co-author) or by MOH workers to evaluate and select volunteer patients for enrollment. The patients ranged from 1 to ≥ 60 years of age and included patients who presented with an acute undifferentiated febrile illness at hospitals, outpatient clinics and private residences in Iquitos. Eligibility for voluntary participation included both males and females who presented with a fever (≥ 38°C) of ≤ 5 days duration, accompanied by a headache, myalgia, and other non-specific signs and symptoms. Only those patients who met the case criteria and who volunteered and provided written informed consent were enrolled. A standardized questionnaire was used by K. B. or an MOH worker to obtain demographic and clinical data for each patient. Also, a venous blood sample was obtained from the arm of each patient during the acute phase of illness, 5 mL from children and 10 mL from adults. When possible, a public health nurse obtained a convalescence blood sample from each patient 14 to 21 days after the onset of illness in their homes or at MOH outpatient clinics. Patient data and blood samples were transported to the NAMRID field laboratories in Iquitos. After clotting, blood samples were separated by centrifugation and aliquots of each patient’s serum was stored at –70°C until shipment on dry ice to the main NAMRID laboratory in Lima for testing for arboviral infections.

### Virus isolation and identification.

Serum samples obtained during the acute phase of illness were tested for arboviruses by inoculation into cell cultures according to standard procedures.^35^ Each sample was thawed and a 1:5 dilution was made in Earle’s based Minimal Essential Medium (EMEM) supplemented with heat inactivated fetal calf serum (FBS) (2%) and streptomycin (200 μg/mL) and penicillin (200 units/mL). Aliquots of 0.2 mL of each diluted sample were inoculated onto each of two confluent medium-free monolayers of VERO cells and two *Ae. albopictus* (C6/36) cell cultures propagated in 25-cm*^2^* flasks. Two of each culture types were inoculated with 200 μL of dilution medium to serve as controls. After Vero cultures were incubated at 37°C and c6/36 at 28°C for 1 hour, 6 mL of EMEM supplemented with 5% FBS and streptomycin and penicillin were added to each culture. Cultures were observed once daily for up to 10 days for cytopathic effects (CPE). If CPE was observed in ∼75% of a culture, aliquots of 1 mL of the remaining cells and culture medium were transferred to sterile 1-dram vials and stored at –70°C. Also, 20-μL aliquots were transferred to each ring of an eight-ringed slide for testing by the indirect fluorescence antibody technique (IFAT).^37^ If CPE was suspected but not clearly evident in some cultures by day 10, the cells were removed from the flasks with a sterile scraper and suspended in the culture medium and passaged directly onto VERO and C6/36 cultures and observed and processed as described for the culture that exhibited CPE.

Viral isolates were identified by the IFAT according to standard procedures.^37^ Briefly, 20-μL aliquots of viral test antigens, viral control antigen, and mock infected cells were added to eight-ringed microscope slides, dried, and then submerged in chilled acetone for 30 minutes. Slides were air-dried at room temperature and stored at –70°C until used in attempts to identify specific viral antigens by fluorescence microscopy. The reference mouse hyperimmune reagents used in the IFAT for preliminary identification of viral isolates included alphavirus, flavivirus, and Simbu grouping antisera obtained from the National Institutes of Allergy and Infectious Diseases. If a suspected viral isolate(s) reacted with a grouping antisera, further IFA testing was performed using flavivirus genus-specific monoclonal antibody 4G2; DENV-1 and -2 monoclonal antibodies 15F3 and 3H5, respectively,^38^ YF monoclonal antibody 2D12,^39^ and Venezuelan equine encephalitis (VEE) virus monoclonal antibody,^40^ and polyclonal hyperimmune mouse ascitic fluids (HMAF) to MAY virus, TR467; ORO TR9760, eastern equine encephalitis (EEE), and VEE subtype 1B 69Z1 viruses, and normal mouse ascitic fluid R143. The HMAF and monoclonal antibodies were used in the IFA at a 1:50 and/or 1:100 dilution. Subtyping of VEE isolates were performed by ELISA using monoclonal antibody and by reverse transcriptase polymerase chain reaction assay as described previously.^40^ Polyclonal hyperimmune mouse serum was used in a plaque reduction neutralization test (PRNT) to identify Bunyaviruses and Caraparu, Guaroa, Group C, Maguari-like, and Murutucu viruses. These reference immune reagents were provided by the UTMB, CDC, and USAMRIID. The hyperimmune ascitic fluids prepared to EEE, VEE, and MAY viruses were used in the IFAT at a 1:50 dilution. Preliminary studies indicated that MAY virus could be differentiated from the other two alphaviruses by IFAT at this dilution. Six of the MAY isolates were confirmed by PRNT.^41^ The isolate from one patient was also sequenced to verify the identity of the virus. Aliquots of the original sera that yielded viral isolates were reinoculated onto cell cultures in attempts to reisolate the viruses. All diagnostic test results were made available either to the patient’s health provider or directly to the patient or to the patient’s parent or guardian.

### Serological assays.

Acute phase of illness serum samples and, when available, convalescent samples obtained 14 to 21 days later were tested together for IgM and IgG antibodies. Confluent monolayers of Vero cells were infected with reference viruses to produce supernatant and lysate antigens to test sera for antibodies as described previously.^31^ A capture ELISA using goat anti-human IgM antibody bound to 96-well Lindbro microtiter plates were used to test for IgM antibody. An indirect sandwich ELISA employing viral-infected or uninfected (controls) cell lysates bound to 96-well microtiter plates was used to test sera for IgG antibody. Sera were screened initially for antibody at a 1:100 dilution, and reactive samples were tested further at a 1:200 through 1:12,800 dilution to determine endpoint titers as described previously. The optical density (OD) value of the content of each microtiter plate well was determined by a spectrophotometer at 414-nm wavelengths. The OD values for the mock antigens were subtracted from those of the viral antigen to yield corrected absorbance values. Serum dilutions with corrected absorbance values greater than the reference cutoff value, estimated as the mean absorbance of 10 antibody negative sera, plus three standard deviations were considered antibody positive. Only the samples with these criteria at a 1:200 dilution were considered antibody positive. The control antibodies for each virus used in the ELISA and the PRNT included mouse hyperimmune ascitic fluid and mouse antisera sera that were provided by the World Reference Center for Emerging Viruses and Arboviruses, Galveston, Texas, and the U.S. Army Medical Research Institute of Infectious Diseases.

The criteria for confirmed cases of VEE, ORO, DEN, and MAY included a febrile illness compatible with the case definition and the isolation of virus and/or a 4-fold or greater increase in ELISA IgM and/or IgG antibody titers between the acute and convalescence sera. A probable case diagnosis was the detection of IgM at a 1:200 dilution or greater in serum samples obtained only during the acute phase of illness. Dengue cases were further classified as presumptive, if samples taken only during the acute phase of illness yielded an IgG antibody titer of 1:25,600 with or without IgM antibody, and patients who had a high (1:25,600 or greater) fixed IgG antibody titer for the acute and convalescent sample. MAY and VEE serological diagnosed cases were distinguished by the IgM antibody. MAY IgM antibody positive cases were either negative for VEE, or the IgM antibody titer was 4-fold or greater for MAY virus. All IgM antibody-positive VEE cases were negative for MAY IgM antibody. All other arboviral cases besides DEN, VEE, ORO and MAY were based on the isolation of virus. The antibody titers were expressed as the reciprocal of the highest dilution yielding a positive value. If positive results were available at the follow-up visit, they were conveyed to the patients.

### Malaria assay.

A drop of blood from each patient was used to prepare a thick smear on a slide, and a second drop was used to prepare a thin smear on a second slide. The smears were stained with Giemsa and examined with a 100× oil immersion objective of a microscope to detect and determine the *Plasmodium* species.

### Assay for leptospiral antibodies.

The microscopic agglutination test (MAT) was used to test for antibodies according to previously published methods, using the standard panel of 23 serovars as described by the U.S. CDC.^42^ A diagnosis of acute leptospirosis was defined as a seroconversion in MAT from negative to 1:100 or greater, 4-fold rise in titer between acute and convalescent sera, or a single titer of 1:800 or greater.

### Statistical methods.

The ages of patients were classified into four age groups (1–14, 15–29, 30–44, ≥ 45 years) for data analysis. Analysis of proportions were compared by the χ^2^ test or Fisher’s exact test. The means of continuous variables were analyzed by Student’s *t* test. When the assumptions of the *t* test were not met, the Mann–Whitney *U* test, a nonparametric rank test, was used to determine whether two samples are likely to derive from the same population. Ninety-five percent confidence intervals (CIs) based on the ratio of two Poisson variables were calculated using the exact conditional distribution. Logistic regression was applied to determine magnitude of associations indicated by odds ratios (ORs) between case status and gender, age groups, occupation, and place.

### Temporal trend analysis.

Temporal trends in the demographic characteristics and prevalence were estimated by fitting a logistic regression model with case status as the dependent variable and year as the independent variable.^43^ By fitting a linear term on the log scale, an estimate of the percentage of average increase or decrease per year was obtained. Analysis for linear trends was performed by testing whether the regression parameter for years was significantly different from zero. All reported CIs are 95%, and all reported *P* values are two-sided; a *P* value < 0.05 was considered statistically significant. All data analyses were performed using SAS for Windows (SAS Institute, Cary, NC).

### Seasonal peak analysis.

Monthly case numbers over each 12-month interval were tested for patterns of variations by Ratchet Circular Scan test for short seasonal peaks.^44^

## RESULTS

### Patients.

Overall, 6,607 febrile patients were studied from October 1, 1993, through September 30, 1999, to determine potential arboviral etiologies of their illness (Table 1). Addresses available for 98.2% (*n* = 6,489) of the patients indicated that most resided in Iquitos city. The number of patients enrolled per year ranged from a low of 262 in 1993 to a high of 2,548 in 1997–1998. Patient enrollment varied per month, with an average of 11 cases for October, November, and December 1993, 25 per month for 1994, 61 in 1995, 43 in 1996, 98 in 1997, 206 in 1998, and 153 in 1999. The distribution of the patients by age and gender is presented in Table 1. The male-to-female ratio was ∼1.5 for each year. The age range was from 1 to 88 years, with a median age of 29 years for females and 26 years for males. Only 4.4% (*n* = 115) of the females and 3.3% (*n* = 132) of the males were older than 60 years. Children, students, and housekeepers were the predominant occupations.

**Table 1:** Temporal trends in the characteristics of all febrile cases, Iquitos, Peru, 1993–1999

Characteristics	Total febrile cases		Period											
	(1993–1999)		1993*		1994		1995		1996		1997		1998	
	*N*	%	*n*	%	*n*	%	*n*	%	*n*	%	*n*	%	*n*	%
No. of patients	6,607	100.0	262	3.9	706	10.7	468	7.1	772	11.7	2,548	38.6	1851	28.0
Gender														
Female	2,645	40.0	101	38.5	291	41.2	178	38.0	184	23.8	1,109	43.5	782	42.2
Male	3,962	60.0	161	61.5	415	58.8	290	62.0	588	76.2	1,439	56.5	1,069	57.8
Age groups (in years)														
1–14	1,000	15.2	53	20.5	121	17.1	70	15.0	100	13.0	403	15.9	253	13.7
15–29	3,183	48.3	105	40.5	296	41.9	228	48.7	523	67.7	1173	46.3	858	46.5
30–44	1,456	22.1	53	20.5	172	24.4	108	23.1	91	11.8	590	23.3	442	23.9
> 44	945	14.4	48	18.5	117	16.6	62	13.2	58	7.5	367	14.5	294	15.9
Mean age (SD)	27.3 (14.8)		27.2 (16.3)		28.2 (15.8)		27.0 (14.8)		22.5 (12.7)		27.6 (14.6)		28.6 (14.9)	
Occupations														
Child/student	1,728	26.2	71	27.1	172	24.3	109	23.3	146	18.9	754	29.6	476	25.7
Housekeeper	1,435	21.7	46	17.6	136	19.3	89	19.0	72	9.3	624	24.5	468	25.3
Agricultural workers	470	7.1	15	5.7	34	4.8	34	7.3	43	5.6	201	7.9	143	7.7
Military personnel	973	14.7	51	19.4	105	14.9	119	25.4	376	48.7	138	5.4	184	10.0
Others†	2,001	30.3	79	30.2	259	36.7	117	25.0	135	17.5	831	32.6	580	31.3
Place of residence														
Iquitos city	5,240	80.8	214	82.6	666	94.3	343	74.9	510	69.8	2175	87.0	1332	72.6
Outside Iquitos city	1,249	19.2	45	17.4	40	5.7	115	25.1	221	30.2	326	13.0	502	27.4

Percents based on total observations. Denominator totals varied slightly due to questionnaire nonresponse.

* Periods: 1993, Oct. 93–Sept. 94; 1994, Oct. 94–Sept. 95; 1995, Oct. 95–Sept. 96; 1996, Oct. 96–Sept. 97; 1997, Oct. 97–Sept. 98; 1998, Oct. 98–Sept. 99.

† Others (workers, merchants, employees, independents, teachers, retired, unemployed).

### Serum samples and etiology of febrile illness.

Of the 6,607 febrile patients studied, blood samples were obtained during the acute and convalescent phases of illness from 57% (*n* = 3,778); samples were available from 43% (*N* = 2,841) only during the acute phase of illness. The overall average number of paired samples obtained during the acute and convalescent phase was 55.2% per year (range 47%–59%), and the average number of samples obtained only during the acute phase of illness was 44.7% per year (range 33%–55%). Assays performed on these samples indicated that 18.8% (*n* = 1,239) of the 6,607 episodes of febrile illness were caused by arboviruses. As shown in Table 2, DENV infection was associated with 967 (14.6%) of the febrile patients, including 712 confirmed and 255 presumptive cases. Only DENV serotypes 1 and 2 were confirmed by virus isolation in cell culture. Other arboviruses included VEEV, subtype ID as the cause of 115 confirmed and 49 presumptive cases (2.5%), OROV as the cause of 19 confirmed and 49 presumptive cases (1.0%), MAYV as the cause of 11 confirmed and 18 presumptive cases (0.4%), and other arboviruses as the cause of 11 cases (0.2%). In addition to arboviruses, tests were performed for malaria on 73% (4,844/6,607) of the febrile patients based on a random sample. The results revealed that 22.9% (*n* = 1,108) were positive, including 16.1% (*n* = 781) for *Plasmodium vivax*, 6.7% (*n* = 322) for *Plasmodium falciparum*, and 0.1% (*N* = 5) were positive for both species. In addition, of 400 patients selected randomly from the negative arbovirus and malaria cases, 9% had serological evidence of leptospirosis.

**Table 2:** Demographic characteristics of arboviral cases in Iquitos, Peru, 1993–1999

Characteristics	Total febrile cases *N*	Dengue cases, *n* (%)	VEE cases *n* (%)	Oropouche cases *n* (%)	Mayaro cases *n* (%)	Malaria cases* *n* (%)
No. of patients	6,607	967 (14.6)	164 (2.5)	68 (1.0)	29 (0.4)	1108 (22.9)
Gender						
Female	2,645	463 (17.5)§	50 (1.9)‡	24 (0.9)	12 (0.5)	359 (18.0)‡
Male	3,962	504 (12.7)	144 (2.9)	44 (1.1)	17 (0.4)	749 (26.3)
Age groups (in years)						
1–14	1,000	114 (11.4)§	26 (2.6)	8 (0.8)‡	2 (0.2)	138 (19.5)
15–29	3,183	443 (13.9)	77 (2.4)	27 (0.9)	16 (0.5)	558 (23.8)
30–44	1,456	240 (16.5)	28 (1.9)	14 (1.0)	4 (0.3)	243 (22.4)
> 44	945	169 (17.9)	33 (3.5)	18 (1.9)	7 (0.7)	167 (24.3)
Occupations						
Child/student	1,728	256 (14.8)§	39 (2.3)‡	17 (1.0)	3 (0.2)	253 (19.5)§
Housekeeper	1,435	269 (18.7)	30 (2.1)	14 (1.0)	9 (0.6)	220 (19.4)
Agricultural workers	470	34 (7.2)	25 (5.3)	6 (1.3)	4 (0.9)	121 (33.6)
Military personnel	973	53 (5.5)	18 (1.9)	5 (0.5)	2 (0.2)	164 (28.9)
Others†	2,001	355 (17.7)	52 (2.6)	26 (1.3)	11 (0.6)	350 (23.7)
Place of residence						
Iquitos city	5,240	877 (16.7)§	123 (2.4)	59 (1.1)‡	21 (0.4)	844 (21.9)§
Outside Iquitos city	1,249	83 (6.7)	41 (3.3)	6 (0.5)	8 (0.6)	247 (27.4)

VEE = Venezuelan equine encephalitis.

* The number of febrile cases tested for malaria was 4,844.

† Others (workers, merchants, employees, independents, teachers, retired, unemployed).

‡ *P* < 0.05.

§ *P* < 0.001.

### Dengue viruses.

Of the 967 DEN cases, 504 (52.1%) were male and 463 (47.9%) were female (gender ratio 1.1:1). However, DENV was more likely to be diagnosed as a cause of disease in female (17.5%) compared with male (12.7%) febrile patients (*P* ≤ 0.001) (Table 2). The number of DEN cases differed significantly among age groups, occupations, and place, with the highest number for persons older than 45 years, housekeepers, and residents of Iquitos city. Cases during the entire study period were significantly older (mean = 30 years) than negative subjects (mean = 27 years) (*P* < 0.001), but not for every year. The mean age of cases from 1993 to 1999 ranged from 24.0 to 33.7 years. Analysis of the case rates by 10 year of age intervals showed a strong linear association with age (*P* < 0.001).

### Venezuela equine encephalitis virus.

The second most common arboviral infection diagnosed was VEE subtype ID, and this was the first association of this virus with cases of human illness in Peru (Table 2). A higher percentage of male febrile patients was diagnosed with VEE compared with the female febrile cases (*P* = 0.012). The gender ratio of VEE cases was 2.3 (95% CI: 1.6–3.3). The case rates differed significantly among occupations but did not differ among age groups (*P* = 0.114) and place (*P* = 0.058). Overall, VEE cases were highest for persons older than 45 years, agricultural workers, and residents outside of Iquitos city. The mean age (28 years) of the VEE cases did not differ significantly from the mean age (27 years) of the VEE-negative patients (*P* = 0.328). The mean age of the cases ranged from 21.0 to 39.5 years. Subjects positive for VEE were not significantly older than negative subjects when analyzed for each study period.

### Oropouche virus.

The third most common arboviral infection was caused by OROV (Table 2). The percentage of the total female cases and male cases diagnosed were not significantly different (*P* = 0.423). The gender ratio of ORO cases was 1.8 in favor of males. Cases differed significantly among the different age groups, as well as by place, but did not differ significantly among occupations (*P* = 0.363). Overall, case rates were highest for persons older than 45, agricultural workers, residents of Iquitos city, and residents of the periurban area. The mean age (31 years) of the ORO cases differed significantly from the mean age (27 years) of the OROV negative patients (*P* = 0.031). The mean age for ORO cases from 1993 to 1998 ranged from 28.1 to 47.0 years.

### Mayaro virus.

MAY was the fourth leading cause of arboviral febrile disease and was the first association of this virus with cases of human illness in Peru (Table 2). Cases were distributed among 12 (41.4%) females and 17 (58.6%) males. The percentage of diagnosed cases by gender was not significantly different (*P* = 0.882). The gender ratio of MAY cases was 1.4 (95% CI: 0.6–3.3) in favor of males. Case rates for MAY did not differ significantly among the study variables. The mean age for MAY cases ranged from 27.7 to 41.2 years from 1995 to 1998. The mean age (33 years) of the MAY cases did not differ significantly from the mean age (27 years) of the MAY negative patients (*P* = 0.058).

### Other arboviral etiologies.

The etiology of an additional 11 (0.2%) of the 6,607 febrile cases included an unidentified Bunyavirus (*n = *1), Caraparu (*n* = 4), Guaroa (*n* = 1), untyped Group C (*n* = 1), Maguari (*n* = 2), Murutucu (*n* = 1), and YF (*n* = 1) viruses. Except for YFV, this was the first association of these viruses with human illness in Peru. All diagnostic test results were based on the isolation of these viruses from serum samples taken during the acute phase of illness. Serological tests were not performed in an attempt to determine whether any of these viruses caused additional cases of human illness. The Bunyavirus case was diagnosed in August 1999 in a 53-year-old male with an unknown occupation. Caraparu virus was identified as the cause of four cases in May 1995 and April and May 1999, including two males (military and general laborer) and two females (student and housekeeper) ranging from 17 to 40 years of age. The Guaroa case was a 30-year-old military employee diagnosed during June 1999. The Group C case was diagnosed in September 1999 and involved a 24-year-old male general laborer. Maguari virus was identified as the cause of one case in January 1999 and involved a 25-year-old male general laborer. Murutucu virus was identified as the cause of two cases during February and March 1999 involving two male (11- and 20-year-old) students. YFV was diagnosed as the cause of one case in January 1998 involving a 19-year-old male fisherman who was referred to Iquitos for medical care from his residence in the Amazon Rainforest.

### Malaria.

Overall, 73.3% (4,844) of the total 6,607 febrile patients were tested for malaria from October 1993 through September 1999. The febrile case infection rate of 22.9% consisted of 781 (16.1%) *P. vivax *and 322* P. falciparum* (6.6%) cases (Table 2). Case counts were significantly different by gender (*P* < 0.05), occupational groups (*P* < 0.001), and place of residence (*P* < 0.001). Malaria cases in 1997 and thereafter were significantly higher among males (26.3%) than females (18.0%; *P* < 0.001). The age of the malaria positive cases (mean = 28 years) did not differ significantly from the malaria negative cases (mean = 27 years; *P* = 0.492). Febrile cases ranging in ages from 15 to 29 years and older than 45 years were more likely to have malaria compared with those febrile cases ranging from 1 to 14 years (*P* = 0.017; *P* = 0.028, respectively). The gender ratio for all malaria cases was 2.1 in favor of males.

### Malaria and arboviral coinfections.

Of the 1,108 positive malaria patients, 12.9% (*n* = 143) were also infected with an arbovirus. DENV was the most common coinfection (6.9%, 76/1,108); however, this percentage was significantly lower than the DEN infection rate (17.0%, 635/3,736) among the malaria-negative cases (*P* < 0.0001). Subjects with both infections were significantly older (mean = 34 years) than subjects infected with only DEN (mean = 28 years; *P *< 0.001). The percent of patients infected with both ORO and malaria was 1.5% (*n* = 17), which did not differ significantly (*P* = 0.150) from the ORO infection rate of 1.0% (*n* = 38) among the malaria negative patients. The mean age (33 years) of patients infected with both malaria and ORO infections did not differ significantly from the mean age for those infected with malaria, but not ORO (mean = 27.6 years) (*P* = 0.101).

VEE was diagnosed in 4.1% (*n* = 45) of the malaria-positive patients, which was significantly greater (*P* = 0.007) than the 2.5% (*n* = 92) of the VEE infections among the malaria-negative patients. There was a significance difference by age among the patients with both infections (mean = 30 years) compared with subjects with malaria and no VEE infection (mean = 27.6; *P* = 0.221). The MAY infection rates among malaria positive 0.5% (*n* = 5) and malaria negative 0.5% (*n* = 17) patients did not differ significantly (*P* = 0.811). However, subjects with both infections were not significantly older (mean = 25.6 years) than subjects with malaria and not MAY infection (mean = 27.7 years; *P* = 0.746).

### Leptospirosis.

Of 400 patients presenting with undifferentiated fever, paired serum samples tested by the MAT showed that 37 (9%) had evidence of acute leptospirosis as supported by seroconversion (4-fold rise in antibody titer), or a single antibody titer ≥ 1/800 for acute serum samples only.

### Arboviral case rates.

Estimates of the number of cases for individual arboviruses per 1,000 febrile cases for each of the 6 years of the study showed that the number did not differ significantly, except that the number of DEN cases for October 1997–September 1998 differed significantly (*P* = 0.001) from those for October 1998–September 1999. When the number of cases were compared between arboviruses by each year, only DEN cases were significantly higher during October 1997–September 1998 and October 1998–September 1999 than for VEE (*P* = 0.005) and for ORO (*P* = 0.010). However, for the overall 6-year period, the number of cases of DEN were significantly higher than for VEE (*P* = 0.001), ORO (*P* = 0.001), and MAY (*P* = 0.017). The number of cases of DEN and VEE increased (*P* = 0.007 and *P* = 0.004, respectively) over the 6-year period, but the number of cases did not increase significantly for ORO and MAY (*P* = 0.464 and *P* = 0.093, respectively).

### Temporal distribution of cases.

As shown in Table 3, time trend analysis controlled for all demographic variables in the multiple logistic regression model showed an increase in the number of DEN cases from 5.7% in 1993 to 18.5% in 1998 and was statistically significant with an average annual change of 6.7% (*P* = 0.007). DEN cases increased for males from 5.6% in 1993 to 18.2% in 1998, with an average annual change of 9.3% (*P* = 0.003), but the average annual change of 3.6% for females was not significant (*P* = 0.616). By age groups, DEN cases increased significantly over the 6-year period among patients who ranged in age from 1 to 14 years (*P* = 0.023) and from 15 to 29 years (*P* = 0.012). Among the different occupations, DEN cases increased significantly only for military personnel (*P* < 0.001). DEN cases increased significantly both within (*P* = 0.008) and outside Iquitos city (*P* = 0.005). For other arboviral infections, VEE cases increased significantly (*P* = 0.004), changes occurred in the number of ORO and MAY but were not significant, and the number decreased significantly for malaria cases (*P* < 0.001) (Table 4).

**Table 3:** Logistic regression and temporal trends analysis of dengue case rates by demographic characteristics, Iquitos, Peru, 1993–1999

Characteristics	Total dengue cases	Periods						Average annual change†	
		1993*	1994	1995	1996	1997	1998	(Oct. 93–Sept. 99)	
	% *N*	% (*n*)	% (*n*)	% (*n*)	% (*n*)	% (*n*)	% (*n*)	%	(95% Cl)
No. of cases	14.6 (967)	5.7 (15)	25.2 (178)	6.2 (29)	2.7 (21)	15.0 (382)	18.5 (342)	6.7§	(1.5–12.1)
Gender									
Female	17.5 (463)‖	5.9 (6)	28.9 (84)	8.4 (15)	4.3 (8)	18.3 (203)‖	18.8 (147)	3.6	(–3.6 to 11.3)
Male	12.7 (504)	5.6 (9)	22.7 (94)	4.8 (14)	2.2 (13)	12.4 (179)	18.2 (195)	9.3§	(2.0–17.2)
Age groups (in years)									
1–14	11.4 (114)‖	3.8 (2)	16.5 (20)‖	2.9 (2)	1.0 (1)	13.2 (53)	14.2 (36)	19.3§	(2.6–38.7)
15–29	13.9 (443)	6.7 (7)	20.9 (62)	5.7 (13)	2.9 (15)	16.3 (191)	18.1 (155)	12.2§	(3.5–21.4)
30–44	16.5 (240)	7.6 (4)	29.7 (51)	8.3 (9)	5.5 (5)	14.4 (85)	19.5 (86)	−0.4	(–9.4 to 9.6)
> 44	17.9 (169)	4.2 (2)	38.9 (45)	8.1 (5)	0 (0)	14.4 (53)	21.8 (65)	−2.2	(–12.2 to 8.9)
Occupations									
Child/student	14.8 (256)‖	4.2 (3)	24.4 (42)‖	7.3 (8)§	2.1 (3)§	16.3 (123)‖	16.2 (77)§	2.1	(–7.8 to 13.0)
Housekeeper	18.7 (269)	6.5 (3)	40.4 (55)	5.6 (5)	2.8 (2)	17.3 (108)	20.5 (96)	−3.7	(–12.4 to 6.0)
Agricultural workers	7.2 (34)	6.7 (1)	11.8 (4)	2.9 (1)	2.3 (1)	5.5 (11)	11.2 (16)	9.9	(–18.6 to 48.4)
Military personnel	5.5 (53)	9.8 (5)	3.8 (4)	0.8 (1)	1.3 (5)	8.0 (11)	14.7 (27)	42.4§	(11.3–82.0)
Others‡	17.7 (355)	3.8 (3)	28.2 (73)	12.0 (14)	7.4 (10)	15.5 (129)	21.7 (126)	2.8	(–5.0 to 11.3)
Place of residence									
Iquitos city	16.7 (877)‖	5.1 (11)	26.6 (177)‖	7.9 (27)§	3.1 (16)	16.1 (350)‖	22.2 (296)‖	6.4§	(1.1–12.0)
Outside Iquitos city	6.7 (83)	8.9 (4)	2.5 (1)	1.7 (2)	5.0 (2.3)	8.6 (28)	8.6 (43)	29.1§	(3.4–61.2)

CI = confidence intervals.

* Periods: 1993, Oct. 93–Sept. 94; 1994, Oct. 94–Sept. 95; 1995, Oct. 95–Sept. 96; 1996, Oct. 96–Sept. 97; 1997, Oct. 97–Sept. 98; 1998, Oct. 98–Sept. 99.

† % Average annual change controlled for all demographic variables in the multiple logistic regression model.

‡ Others (workers, merchants, employees, independents, teachers, retired, unemployed).

§ *P* < 0.05

‖ *P* < 0.001

**Table 4:** Logistic regression and temporal trends analysis of arboviral cases, Iquitos, Peru, 1993–1999.

Arboviral cases	Total cases	Periods						Average annual change†	
		1993*	1994	1995	1996	1997	1998	(Oct. 93–Sept. 99)	
	% (*N*)	% (*n*)	% (*n*)	% (*n*)	% (*n*)	% (*n*)	% (*n*)	%	(95% Cl)
Dengue	14.6 (967)	5.7 (15)	25.2 (178)	6.2 (29)	2.7 (21)	15.0 (382)	18.5 (342)	6.7§	(1.5–12.1)
VEE	2.5 (164)	6.1 (16)	0.4 (3)	0.4 (2)	1.2 (9)	2.8 (70)	3.5 (64)	18.1§	(4.3–33.6)
Oropouche	1.0 (68)	0 (0)	0.6 (4)	0.6 (3)	0.9 (7)	1.8 (47)	0.4 (7)	11.6	(–7.1 to 34.1)
Mayaro	0.4 (29)	0 (0)	0 (0)	0.4 (2)	0.8 (6)	0.6 (15)	0.3 (6)	12.5	(–15.5 to 46.9)
Malaria‡	22.9 (1108)	11.3 (16)	27.3 (50)	38.9 (32)	38.7 (115)	25.8 (648)	15.2 (247)	−12.1‖	(–17.0 to −6.8)

CI, confidence intervals; VEE, Venezuelan equine encephalitis virus.

* Periods: 1993, Oct93-Sep94; 1994, Oct94-Sep95; 1995, Oct95-Sep96; 1996, Oct96-Sep97; 1997, Oct97-Sep98; 1998, Oct98-Sep99.

† % Average annual change controlled for all demographic variables in the multiple logistic regression model.

‡ The number of febrile cases tested for malaria was 4844.

§ *P* < 0.05

‖ *P* < 0.001

### Seasonal variation of cases.

**Figure 2.**
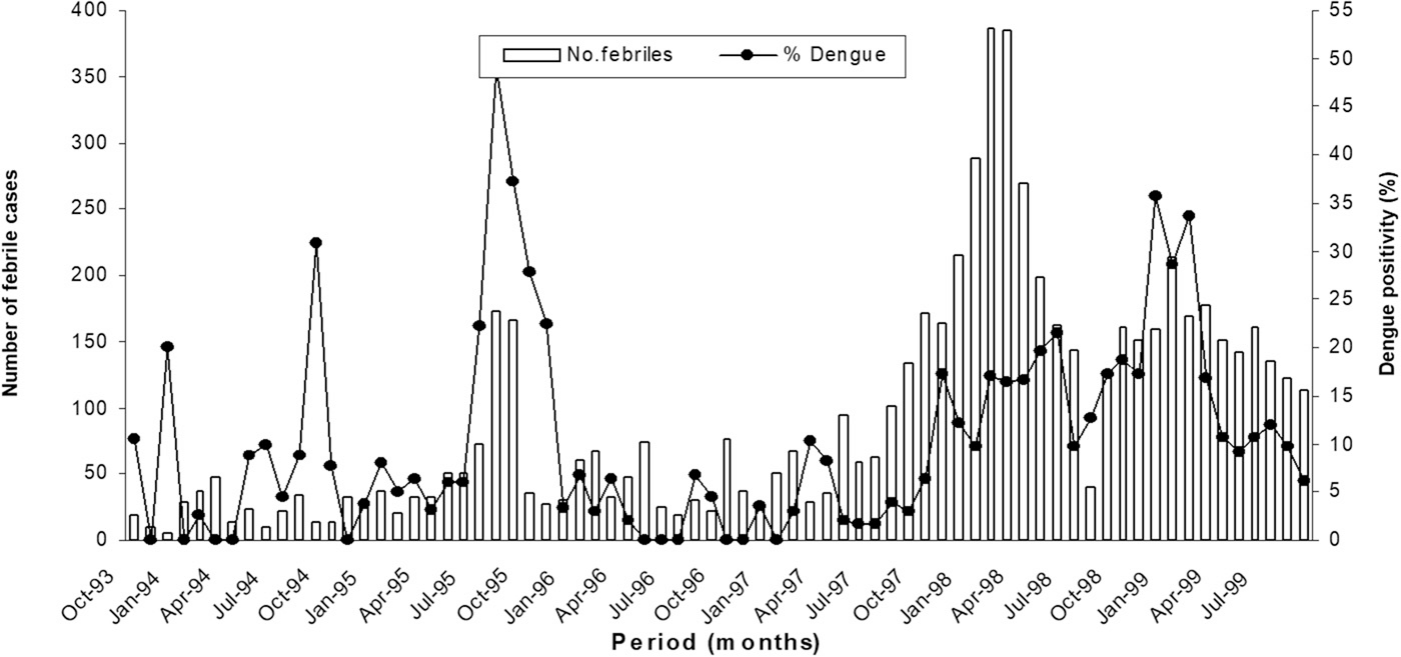
Temporal distribution of dengue cases as a percent of the total number of febrile cases.

There were short transient seasonal peaks for DEN, ORO, VEE, and MAY. However, the seasonal months were different for each virus. The temporal distribution of DEN cases as a percentage of the total febrile patients is presented in Figure 2. The highest seasonal peak for DEN occurred from January to March (34% of all cases; *P* = 0.005). ORO cases occurred more frequently from November through January (47%) than in other months (*P* = 0.005). VEE and MAY cases peaked from February to April (40%; *P* < 0.001 and 50%; *P* < 0.025, respectively)

### Clinical signs and symptoms.

The frequency of clinical signs and symptoms associated with the DEN, VEE, ORO, and MAY cases are summarized in Table 5. Clinical observations were collected at the time the patients were enrolled and when the convalescent blood samples were obtained. None of the patients presented with encephalitis or hemorrhagic manifestations, and there were no fatalities. Eye pain (85%), arthralgia (89%), rash (25%), nausea/vomiting (55%), and chills (91%) were significantly more frequent among the DEN cases than the patients who were negative for DEN (*P* < 0.01). Only nausea/vomiting (59.8%) were significantly more frequent among the VEE cases (*P* = 0.036). Cough (23.9%) was significantly more frequent among ORO patients (*P* = 0.046) and a rash (37.9%) was more common but not significantly different among MAY patients (*P* = 0.11) than negative patients. Overall, only the frequency of chills, eye pain, headache, and rash differed significantly among the four viruses. DEN patients had a significantly higher percentage of chills (*P* = 0.029) and rash (*P* < 0.001) than VEE patients. Eye pain (*P* < 0.001) was significantly more frequent among DEN than ORO patients, and headache (*P* < 0.001) and chills (*P* = 0.013) were significantly more frequent among DEN than MAY patients. Eye pain (*P* = 0.019) was significantly more frequent among VEE patients than ORO patients, and headache (*P* < 0.001) was more common among VEE cases than MAY, and rash (*P* < 0.001) more common among MAY cases than VEE cases. Among the ORO patients, the frequency of rash (*P* = 0.025) was lower than that for MAY, and headaches (*P *< 0.001) occurred more frequently than among the MAY patients.

**Table 5:** Clinical signs and symptoms for febrile cases, Dengue, Venezuelan equine encephalitis, Oropouche and Mayaro cases in Iquitos, Peru, 1993–1999

Signs/Symptoms	Febrile cases		Dengue cases		VEE cases		Oropouche cases		Mayaro cases	
	(*N* = 6,607)		(*N* = 967)		(*N* = 164)		(*N* = 68)		(*N* = 29)	
	*n*	%	*n*	%	*n*	%	*n*	%	*n*	%
Fever	6,438	97.4	947	98.3	162	98.8	66	98.5	28	96.6
Headache	6,392	96.7	945	97.7	160	97.6	63	94.0	29	10.0
Eyes pain	5,357	81.1	818	84.6*	137	83.5	46	68.7	24	82.8
Body pain	6,014	91.0	896	92.7	155	94.5	59	88.1	27	93.1
Arthralgia	5,635	85.3	859	89.0*	145	88.4	56	83.6	26	89.7
Rash	1,078	16.3	244	25.3†	15	9.2	10	14.9	11	37.9†
Nausea/vomiting	3,406	51.6	535	55.3*	98	59.8*	35	52.2	14	48.3
Diarrhea	1,590	24.1	213	22.1	37	22.6	18	26.9	2	6.9
Chills	5,803	87.8	880	91.2*	140	85.4	61	91.0	22	75.9
Cough	2,582	39.1	303	31.4	45	27.4	16	23.9*	13	44.8
Nasal congestion	1,353	20.5	146	15.2	16	9.8	10	14.9	4	13.8
Sore throat	1,564	23.7	185	19.1	27	16.5	18	26.9	6	20.7

Denominators total varied due to questionnaire nonresponse.

* *P* < 0.05.

† *P* < 0.001.

The severity of illness caused by other arboviruses varied from mild to moderate and ranged in duration from 1 to 6 days. The prominent clinical signs and symptoms for all the cases were fever, headache, chills, eye and body pain, arthralgia, sore throat, and nasal congestion. The only deviation was that three of the four Caraparu cases also presented with nausea and vomiting.

### Risk factors.

Characteristics associated with arboviral infections are shown in Table 6. Overall, multivariate logistic analysis, after controlling for gender and age, indicated that the risk of DEN was greater among females (*P* < 0.001). Being ≥ 15 years old was significantly associated with DEN, compared with ≤ –14 years (15–29 years: *P* = 0.007; 30–44 years: *P* < 0.001; ≥ 45 years: *P* < 0.001). The risk of contracting DENV infection was significantly greater among residents of Iquitos city (*P* < 0.001). Of the different arboviruses, only VEE posed a significant risk to agricultural workers (*P* = 0.009). For ORO, the risk was greater among patients who were 45 years or older (*P* = 0.041) and for patients who resided in peri-urban locations (*P* = 0.029). No significant associations were found for MAYV infections.

**Table 6:** Characteristics associated with arbovirus infections in febrile cases, Iquitos, Peru, 1993–1999

Characteristics	Dengue OR* (95% Cl)	VEE OR (95% Cl)	Oropouche OR (95% Cl)	Mayaro OR (95% Cl)
Gender (male)				
Female	1.4 (1.2–1.6)§	0.6 (0.5–0.9)	0.8 (0.5–1.3)	1.0 (0.5–2.1)
Age groups (1–14) (in years)				
15–29	1.4 (1.1–1.7)‡	0.9 (0.5–1.3)	1.0 (0.5–2.3)	2.6 (0.6–11.3)
30–44	1.6 (1.2–2.0)‡	0.7 (0.4–1.2)	1.2 (0.5–2.9)	1.4 (0.3–7.5)
> 44	1.7 (1.3–2.2)‡	1.4 (0.8–2.3)	2.4 (1.1–5.6)‡	3.7 (0.8–17.9)
Occupations (child/student)				
Housekeeper	0.9 (0.7–1.2)	1.4 (0.7–2.7)	0.8 (0.3–2.0)	4.2 (0.8–20.9)
Agricultural workers	0.3 (0.2–0.5)	2.4 (1.2–4.5)§	0.7 (0.2–2.1)	4.5 (0.8–25.7)
Military personnel	0.3 (0.2–0.5)	0.7 (0.4–1.2)	0.4 (0.1–1.1)	1.1 (0.2–6.7)
Others†	1.0 (0.8–1.2)	1.1 (0.7–1.8)	0.8 (0.4–1.7)	2.9 (0.7–12.3)
Place of residence (out Iquitos city)				
Iquitos city	2.7 (2.1–3.4)§	0.7 (0.5–1.1)	2.3 (0.9–5.4)	0.6 (0.3–1.3)

CI = confidence interval; VEE = Venezuelan equine encephalitis.

* Odds ratio adjusted for gender and age in years. The categories in parenthesis describe the baseline group for odds ratio calculation.

† Others (workers, merchants, employees, independents, teachers, retired, unemployed).

‡ *P* < 0.05.

§ *P* < 0.001.

## DISCUSSION

The results of our 6-year study are among the most extensive descriptions of the arboviral etiologies of febrile illnesses in the Amazon basin. Overall, 18.7% of the 6,607 febrile patients studied were caused by one or more arboviruses, 22.9% of 4,844 were positive for either *P. falciparum *or* P. vivax*, and 9% of 400 patients had evidence of leptospirosis. Although the study was not designed to assess the importance of these pathogens as a cause of human morbidity in the population, the findings indicated that arboviruses, leptospirosis, and malaria were common causes of acute febrile illnesses. A more quantitative assessment of their importance was precluded because estimates were not determined of the overall total number of febrile cases in the population as specified by the case definition. However, we believe that the data were representative of the Iquitos population because of the continuous enrollment of patients of all age groups over a 6-year period employing a standardized case definition at most of the health care clinics and hospitals. Although the percentage of the total febrile cases enrolled was unknown, the systematic approach of continuous sampling of patients from 1993 to 1999 made it possible to gain an understanding of some of the more frequent arboviral illnesses and allowed for a valid comparison of demographic and clinical data and to infer temporal relationships of the etiology of cases and associated risk factors. Finally, the results are important for understanding the relative health burden of these diseases and to serve as baseline knowledge for designing and implementing further study and for assessing the health threat of these pathogens.

Overall, 11 arboviruses were identified as the causes of the febrile cases, including DENV, VEEV, OROV, MAYV, an untyped Bunyavirus, Caraparu, Guaroa, an untyped Group C virus, Maguari, Murutucu, and YFV. The diagnosis of cases of DEN, VEE, ORO, and MAY cases were based on both virus isolations and serological results. The cases associated with the other viruses were diagnosed by virus isolations alone and therefore were not included in the overall analysis. Among the DEN cases, virus was isolated from 192, and the other 775 were diagnosed by serological results. In total, 47 cases of VEE were diagnosed by virus isolations, and 117 cases were based on serological results. The ORO cases included four that were diagnosed by virus isolation and 64 by serological results. Of the MAY cases, six were based on virus isolations and 23 on serological results. The possibility that the serological results for dengue represented infection by related viruses in South America, such as YFV, Ilheus, and ROC was not considered likely.^15^ Only one case of YF was diagnosed by virus isolation. This case was not from Iquitos proper, nor were there any YF vaccine campaigns in Iquitos during the study period. Ilheus virus was not isolated from any of the patients, even though the virus was obtained from mosquitoes during an ongoing separate study in the rainforest near Puerto Almendras about 20 km Southwest of Iquitos.^45^ Thus far, evidence of ROC virus has not been reported from the Iquitos area. Only VEEV and MAYV of the *Alphavirus* genus were isolated from the febrile cases. MAY and VEE serological diagnosed cases were distinguished by the specificity of the IgM antibody. MAYV IgM antibody positive cases were either negative for VEE, or IgM antibody titer was 4-fold or greater for MAYV, but not for VEEV, and all IgM antibody–positive VEE cases were negative for MAYV IgM antibody. Although other alphaviruses were not detected, numerous isolates of EEE virus (now classified in the species *Madariaga* for South American strains) and Una viruses were obtained from mosquitoes during the nearby study in Puerto Almendras.^46^ The low frequency of viral isolations and high percentage of serologically diagnosed ORO cases is not understood. This finding differs markedly from during studies in Brazil that showed OROV could be isolated from 72% of the patients on the third day of illness and 44% on the fourth day.^47^ Consideration was given to the possibility that another Simbu-related virus may have been causing infections and was being missed by the hyperimmune OROV antiserum used in our immunofluorescence assays. If so, the serological data could then represent cross-reactivity with another Simbu group virus. OROV is the only known member to cause human disease in South America.^15^ Jatobal virus, a member of the Simbu group virus, is considered to be a reassortant that contains the small RNA of OROV.^48^ The only isolate of this virus in South America was obtained during 1985 from a carnivore in Para state, Brazil, and thus far has not been associated with human infection. Utinga virus, the only other member of the Simbu group, was only isolated from a three-toed sloth (*Bradypus tridactylus*) in Brazil and has not been associated with human disease.^15^

The approach used to diagnose the febrile cases was to test sera in VERO cells because of their susceptibility to many of the known arboviruses. In addition, infection is usually readily demonstrable microscopically because most arboviruses produce a cytopathic effect (CPE) in Vero cells. The *Aedes albopictus* (C6/36) cells are also highly susceptible, but infection, especially with DEN viruses, is not usually cytolytic, and therefore, CPE is not a reliable indicator of infection. Consequently, all C6/36 cell cultures without evidence of CPE were screened for evidence of DENV infection by IFA. Newborn mice were also used in an attempt to isolate viruses that may not have been detectable by cell cultures. Although the data were not analyzed, some isolates were obtained by only one assay method. The use of multiple assays was labor intensive, but the high diagnostic success rate was clearly attributable to this approach. The serological approach of using IgM antibody was effective but not always applicable because of the unavailability of a convalescent sample for almost half of the cases. Although arboviruses, *Plasmodium *spp. and *Leptospira* were the likely cause of some of the remaining undiagnosed cases, several other viral, bacterial, and parasitic illnesses, such as typhus, measles, rubella, enteroviruses, influenza, hepatitis A and B, typhoid, and others are also known to cause acute febrile illnesses in the Iquitos area.

Our results underscore the importance of the application of a laboratory diagnosis to determine the specific etiologies of febrile illnesses. Diagnostic tests for most viral infections are not available in public health laboratories in Iquitos for routine testing, and thus cases that met the clinical definition used in this study would have been diagnosed solely on the basis of clinical signs and symptoms. As a result, such cases in Iquitos would probably have been recorded by the MOH as being caused by either malaria or dengue viruses. Although these pathogens were the cause of a high percentage of the cases, other arboviruses, as well as leptospires, were shown to be the cause of a substantial number of the cases. The misdiagnosis of cases based solely on a clinical syndrome is likely to be widespread as documented by a recent report from Belem Brazil that showed a case diagnosed clinically as dengue to have been caused by cytomegalovirus.^49^ Also, cases observed during an outbreak of rubella based on clinical observations were found by laboratory testing to have been caused by MAYV and YFV.

The results of this study and previous observations indicated that dengue was the most commonly diagnosed arboviral disease in the urban community of Iquitos, with case rates ranging from a low of 2.7% in 1996 to a high of 25.2% in 1994.^21^^,^^34^^,^^35^ The clinical manifestations were confined to a febrile illness, characterized more commonly by a fever, chills, headache, ocular and generalized body pain, nausea and vomiting, and arthralgia without hemorrhagic disease, even though estimates indicated that as many as many as 10,000 secondary infections involving DEN-1 followed by DEN-2 occurred in 1995.^35^ Cases were significantly higher among patients 15 to > 45 years of age and among females. This age distribution pattern for the outbreaks of dengue was likely to reflect the introduction of this virus into a virgin population, rather than a lower susceptibility among children. In addition, cases among children tended to very mild, and therefore they may have been less likely to seek medical care.^35^ The higher rates among females were consistent with previous observations for DEN cases, given that *Ae. aegypti* are common in the peridomicilliary environment. The occupational groups affected most, including housekeepers, children, students, and others, can be attributed to a more urban lifestyle, in contrast to military and agricultural workers who frequently lived and worked outside the city. Dengue cases occurred throughout the year, but the highest seasonal peak occurred from January to March (34% of all cases). This temporal pattern parallels the rainy season and a corresponding increase in the *Ae. aegypti* population (A. C. Morrison, unpublished data). However, studies on the relation of climate to dengue cases have shown weak, direct relationships between climatic variables and total dengue cases implying that there were other undefined factors that determined the temporal patterns of DENV transmission in Iquitos.^50^^,^^51^ The growing importance of DEN as a public health problem in Iquitos was supported by annual increasing pattern among the 1 to 27 years of age patients with rate ranging from 5.7% in 1993 to 18.5% in 1998, or an average increase of 6.7% per year.

Although an enzootic pattern of VEEV subtype ID transmission has been reported, our data are the first documentation of suggested enzootic transmission in a human population.^52^^,^^53^ The VEE subtype D cases were characterized by a self-limited acute febrile illness, with features similar to those reported previously and to those reported here for DEN cases, except for a much lower frequency of rash. Although VEEV subtype ID has been reported to cause severe and fatal cases of encephalitis, none of the cases presented with any evidence of central nervous system signs or symptoms.^9^^,^^54^ In addition, the number of cases did not differ significantly by age group but were significantly more common among males and those living outside Iquitos. The comparable case rates by age group did not reflect an increasing pattern with age as would be expected for an enzootic transmission of the virus. Thus, this pattern may represent the likely spread of the virus to segments of the population that had not been previously exposed to the VEE virus. The risk of infection was also significantly more common among agricultural workers and therefore may account for the higher rates among males. VEE cases occurred throughout the year but peaked from February to April, with an overall significant decline in cases over the study period. The pattern of sporadic occurrence and low number of cases is not understood but is typical of the epidemiological characteristics of VEEV subtype 1D infection of humans in rural and forested areas.^9^

The findings of this study supported an enzootic pattern of OROV transmission, with case rates occurring more frequent in Iquitos between November through January (47%).^55^ Overall, the clinical presentations were typical of the febrile clinical syndrome described for cases in Brazil.^10^^,^^56^ However, the pattern of enzootic transmission of OROV in Iquitos differed from that in Brazil where transmission was characterized by explosive outbreak affecting more than 100,000 people in small and large urban communities. In contrast to only febrile cases described in Iquitos, some of the cases in Brazil relapsed with original signs and symptoms within the first 10 days after the onset of illness, and some cases presented with a rash and with severe but nonfatal meningitis symptoms. The differences in clinical presentations led to studies that showed the Iquitos OROV to differ genetically from those isolated in Brazil, but the significance, if any on disease outcome is unknown.^57^ The cases in Iquitos were more frequent among the 15 to > 45 years of age, with a higher percentage among males and agricultural workers and with significantly more cases in Iquitos. Studies in Brazil showed that the male-to-female ratio cases varied in some outbreaks and not in others and that all ages were affected, but rates did not differ significantly by age groups.^56^

Clinically, the MAY fever cases in Iquitos were similar to those reported elsewhere in South America.^11^^,^^41^^,^^58^^–^^60^ Cases peaked during the rainy season, or from February through April. The more prominent signs and symptoms were fever, chill, headache, ocular and body pain, arthralgia, rash, nausea and vomiting, and cough. As described previously, arthralgia persisted for 2 months or more for some of the patients.^59^^,^^60^ More cases occurred among males and were more common among patients ranging in age from 15 to > 45 years. Although population-based infection rates in Iquitos were unknown, this age distribution pattern involving the older age groups was suggestive of a recent introduction of MAYV into the population. More than half of the MAY cases occurred in Iquitos and therefore extended the risk of this disease to this urban population.

Our findings that febrile cases in Iquitos were caused by an unidentified Bunyavirus and by Caraparu, Guaroa, untyped Group C, Maguari, and Murutucu viruses further extends the number of arboviruses associated with human disease in Peru.^61^^,^^62^ The data on relative importance and the frequency of cases in Iquitos were limited because a serological diagnosis was not attempted for any of the cases. However, studies conducted in other countries of South America provide an overview of existing knowledge on the ecology and epidemiology of these viruses.^15^^,^^62^ Our finding of these viruses as a cause of human disease in an urban community is contrary to the well-documented associations with cases in or near the rainforest. The apparent adaptation of Caraparu, Guaroa, Maguari, and Murutucu viruses to an urban community further emphasizes the growing importance of these arboviruses as a threat to human health and therefore warrant further studies to elucidate their ecology and epidemiology.

The observations that *P. falciparum* and *P. vivax* were causes of febrile illnesses in Iquitos were consistent with previous findings.^63^ However, *P. falciparum* was only recently reintroduced at the time of this study into the Iquitos area, and as supported by our results, has in only a few years increased markedly. Although only a small sample of the total cases was tested for *Leptospira* in our study, the more recent findings indicated that leptospirosis was a common cause of human disease, including the association of a new *Leptospira *spp., *L. licerasiae*, as a cause of human disease.^64^ The frequency of coinfected cases involving malaria and arboviruses are among the highest reported for these pathogens. This observation has important public health implications in that arbovirus cases are likely to be underestimated because most laboratories are equipped to perform only diagnostic tests for malaria. These data document the recent increase and spread of *P. falciparum* into Iquitos and is an additional example of the growing spread and health burden that arthropod-borne pathogens are having on the urban population in Peru. It likely represents the evolving pattern of malaria in other urban communities in the Peruvian Amazon.

Because the data were generated by this study from 1993 to 1999, the impact of arboviral and other vector-borne diseases on the health of the Iquitos population and the development of disease prevention strategies for dengue has been described in several additional studies.^65^^–^^83^ Among these studies, the most comprehensive investigation was conducted from 1999 until the present on the epidemiology of DENV and associated diseases and the ecology and control of *Ae. aegypti*, the vector of these viruses. The study most relevant to the present investigation was a comprehensive study conducted from 2000 to 2007 that determined the etiologies of febrile illnesses at 13 locations in Ecuador, Peru, Bolivia, and Paraguay.^66^ Among 20,880 febrile patients included in that study, evidence of a recent arbovirus infection was detected for 6,793 (32.5%) patients. As observed in our study, the most common arbovirus infection was dengue, making up 26.0% of total number of cases. Other arboviruses caused about 3% of the febrile cases, including VEEV and MAYV and OROV, and Group C viruses. Our studies only in Iquitos, as mentioned earlier, from 1993 to 1999 enrolled 6,607 patients in and around this urban community, whereas the subsequent study enrolled 10,739 patients in and around Iquitos. During the subsequent study, DENV was the most common cause of febrile cases in Iquitos, followed by VEEV, OROV, MAYV, and Group C viruses.^66^ The incidence of DENV cases varied, peaking in 2002 with 554.0/100,000 following the introduction of DENV-3 and averaging 274.7/100,000 over the 7-year period. The average incidence for other predominant arboviruses were much lower, including 28.1/100,000 for VEEV, 8.5/100,000 for MAYV, 14.3/100,000 for OROV, and 14.2/100,000 for Group C viruses.^66^ Also, this subsequent study in Iquitos suggested that malaria caused approximately 30% of acute febrile illnesses. Overall, the approach used in this subsequent study from 2000 to 2007 was similar to our study, except that our enrollment age ranged from 1 year to ≥ 60 years of age, whereas the subsequent study enrolled patients 5 years of age or older. The viruses, including DENV, VEEV, OROV, MAYV, and Group C viruses were reported as the cause of febrile cases in this subsequent study were the same are those observed during our study. However, in the present study, additional viruses, including an untyped Bunyavirus, Caraparu, untyped Group C, Maguari, and Murutucu viruses, were associated with febrile cases. Only DEN-1 and -2 were reported as the cause of febrile cases in our study, whereas all four DEN serotypes were associated with cases in the subsequent study with DENV-3 being introduced into the Iquitos community in 2001 and DENV-4 during 2008.^66^^,^^83^ Malaria was reported as the cause of febrile cases in both studies and antibody was detected to leptospiral infection in a subsample of cases in the present study suggested that febrile illnesses were caused by this pathogen, but cases were not tested for leptospiral infection as a possible cause of cases during the subsequent study. In addition to the comprehensive studies that described the arbovirus etiology of febrile illnesses in Ecuador, Peru, Bolivia, and Paraguay,^66^ the little-known, obscure Guaroa virus was identified during 2010 as the cause of febrile illness in Iquitos and surrounding periurban and rural areas.^84^^,^^85^ In 2011, Iquitos virus, a novel reassortant orthobunyavirus; in 2015, Itaya, a novel virus; and in 2016, Zika virus were first associated with human febrile illnesses.^86^^–^^89^ Other vector-borne pathogens reported to be a cause of febrile illnesses in the Iquitos community include the spotted fever and typhus *Rickettsia *groups.^90^^,^^91^

Arboviruses are among the most common cause of human febrile illness worldwide and continue to emerge as the cause of global epidemics.^1^^,^^66^ As reported in this and subsequent studies,^66^ the findings provide a better understanding of the impact arboviruses as the cause of febrile illnesses in Iquitos, Peru, an urban community in the Amazon Rainforest. In addition, the isolation and identification of arboviruses, including eastern equine encephalomyelitis (*Madariaga*), Trocara, Una, Venezuelan equine encephalomyelitis, western equine encephalomyelitis viruses, Ilheus, St. Louis encephalitis, Caraparu, Itaqui, Mirim, Murutucu, Wyeomyia, and El Huayo viruses from mosquitoes in Puerto Almendras, Peru approximately 20 km west-southwest of Iquitos., together with the arboviral causes of febrile illnesses described in this and a subsequent study is among the most comprehensive description of arboviruses in the Americas.^45^^,^^66^^,^^92^ Although significant progress has been made in understanding the ecology and epidemiology of DENV in the Iquitos community, little is known about the ecology and epidemiology of the many other arboviruses and potential arthropod vectors. The lack of this critical information represents a major knowledge gap and underscores the importance of continued surveillance to identify new and known arboviruses and to be prepared to respond effectively to the possibility that one or more of these viruses will emerge to cause the next global pandemic.^93^^,^^94^
